# Effect of Vasoactive Intestinal Polypeptide on Development of Migraine Headaches: A Randomized Clinical Trial

**DOI:** 10.1001/jamanetworkopen.2021.18543

**Published:** 2021-08-06

**Authors:** Lanfranco Pellesi, Mohammad Al-Mahdi Al-Karagholi, Roberto De Icco, Hande Coskun, Fatima Azzahra Elbahi, Cristina Lopez-Lopez, Josefin Snellman, Jens Hannibal, Faisal Mohammad Amin, Messoud Ashina

**Affiliations:** 1 Danish Headache Center, Department of Neurology, Rigshospitalet Glostrup, Faculty of Health and Medical Sciences, University of Copenhagen, Copenhagen, Denmark; 2 Headache Science & Neurorehabilitation Center, Istituto di Ricovero e Cura a Carattere Scientifico Mondino Foundation, Pavia, Italy; 3 Department of Brain and Behavioral Sciences, University of Pavia, Pavia, Italy; 4 Novartis Pharma AG, Basel, Switzerland; 5 Department of Clinical Biochemistry, Bispebjerg Frederiksberg Hospital, Faculty of Health Sciences, University of Copenhagen, Copenhagen, Denmark

## Abstract

**Importance:**

Vasoactive intestinal polypeptide (VIP) and pituitary adenylate cyclase-activating polypeptides (PACAPs) are structurally and functionally related, yet different in their migraine-inducing properties. It remains unclear whether the lack of migraine induction can be attributed to the only transient vasodilatory response after a 20-minute infusion of VIP.

**Objective:**

To determine whether a 2-hour infusion of VIP would provoke migraine attacks.

**Design, Setting, and Participants:**

A randomized, double-blind, placebo-controlled, crossover study was conducted between May and September 2020 at the Danish Headache Center in Copenhagen, Denmark. Patients were eligible for inclusion if they were ages 18 to 40 years, weighed between 50 and 90 kg, had a diagnosis of migraine without aura as defined by the International Classification of Headache Disorders, and had a migraine frequency of 1 to 6 attacks per month.

**Interventions:**

Patients were randomly allocated to receive a 2-hour infusion of VIP or placebo on 2 different days.

**Main Outcomes and Measures:**

The primary end point was the difference in incidence of experimentally induced migraine attacks during the observational period (0-12 hours) between VIP and placebo.

**Results:**

Twenty-one patients (17 [81%] women and 4 [19%] men; mean [range] age, 25.9 [19-40] years) were recruited in the study. Fifteen patients (71%; 95% CI, 48%-89%) developed migraine attacks after VIP compared with 1 patient (5%; 95% CI, 0%-24%) who developed a migraine attack after placebo (*P* < .001). The VIP-induced migraine attacks mimicked patients’ spontaneous attacks. The area under the curve (AUC) of headache intensity scores (0-12 hours), as well as the AUC of the superficial temporal artery diameter (0-180 minute) were significantly greater after VIP compared with placebo (AUC_0-12h_, *P* = .003; AUC_0-180min_, *P* < .001).

**Conclusions and Relevance:**

A 2-hour infusion of VIP caused migraine attacks, suggesting an important role of VIP in migraine pathophysiology. VIP and its receptors could be potential targets for novel migraine drugs.

**Trial Registration:**

ClinicalTrials.gov Identifier: NCT04260035

## Key Points

**Question:**

Can vasoactive intestinal polypeptide (VIP) cause migraine attacks?

**Findings:**

In this crossover study of 21 patients with migraine without aura, a 2-hour infusion of VIP provoked migraine in 15 patients (71%) compared with 1 patient who experienced a migraine after placebo (5%).

**Meaning:**

These findings suggest that the role of VIP or a prolonged dilation of cranial arteries might be critical in migraine initiation.

## Teaser

This randomized clinical trial examines whether a 2-hour infusion of vasoactive intestinal polypeptide provokes migraine attacks.

## Introduction

Migraine is a neurovascular disorder with biological underpinnings that involve a complex interplay of the trigeminovascular system and deep brain structures.^1^ The trigeminovascular system consists of autonomic efferent projections and perivascular trigeminal afferent neurons that innervate cranial blood vessels with nociceptive signals to central trigeminal neurons in the spinal trigeminal nucleus.^2^ Upon activation of the trigeminovascular system, afferent and efferent nerve fibers release various vasoactive peptides, which may be involved in the initiation of a migraine attack.^2,3,4^ Vasoactive intestinal polypeptide (VIP) and pituitary adenylate cyclase-activating polypeptides (PACAPs) are structurally related members of the VIP/secretin/glucagon superfamily of peptides.^5^ After being released,^6^ these peptides stimulate G-protein coupled receptors (GPCRs) and initiate downstream signaling cascades in proximity to the cranial vessels, involving cyclic adenosine monophosphate (cAMP).^7,8^ Experimental studies have demonstrated that PACAP isoforms (PACAP27 and PACAP38) cause migraine attacks after a 20-minute infusion in patients with migraine without aura.^9,10,11^ Furthermore, provoked migraine attacks were accompanied by a 4-hour lasting dilation of the superficial temporal artery (STA) and the middle meningeal artery (MMA).^12,13^ In contrast to PACAPs, 20-minute infusion of VIP only resulted in a short-lasting dilation of the STA and the MMA^10,14^ and no attacks in 1 study^15^ or 4 attacks in 22 patients in another study.^10^ Of note, experimental studies in humans demonstrated that a prolonged vasodilation of extracranial arteries leads to transient structural changes in the arterial wall and triggers a throbbing headache associated with nausea.^16,17,18^ VIP is the only peptide that causes a short cranial arterial dilation and no migraine. To date, it remains unclear whether the limited migraine-inducing property of VIP can be attributed to its equally limited vasodilatory response. Recently, we developed a model of 2-hour infusion of VIP in healthy volunteers, which caused a prolonged dilation of STA and delayed headache.^19^

We hypothesized that prolonged intravenous infusion of VIP would provoke migraine attacks in patients with migraine without aura. To test this hypothesis, we conducted a randomized, double-blind, placebo-controlled, 2-way crossover study.

## Methods

The study was approved by the Ethics Committee of Copenhagen and the Danish Data Protection Agency and was conducted according to the Declaration of Helsinki of 1964.^20^ All patients provided written informed consent before inclusion. This study follows the Consolidated Standards of Reporting Trials (CONSORT) reporting guideline. The trial protocol is available in Supplement 1.

### Recruitment of Patients

We recruited 21 otherwise healthy male and female patients with migraine without aura. All patients were recruited from the Danish Headache Center or through the Danish test subject website.^21^ Patients were eligible for inclusion if they were aged 18 to 40 years, weighed between 50 kg and 90 kg, had a diagnosis of migraine without aura as defined by the International Classification of Headache Disorders (ICHD-3),^22^ and had a migraine frequency of 1 to 6 attacks per month. Women who used oral contraceptives or an intrauterine device were included in this study. Exclusion criteria included any other type of headache (including having more than 3 days of tension-type headache per month) as defined by the ICHD-3; anamnestic or clinical signs of cardiovascular disease; previous serious somatic or psychiatric diseases; substance abuse or smoking; daily intake of any medicine apart from oral contraceptives, including prophylactic migraine treatment; and women who were pregnant or breastfeeding. A full medical examination was performed on the day of recruitment. Patients were informed that VIP might induce headache in some individuals, but the timing or the characteristics of headache were not discussed.

### Experimental Design

In a double-blind, randomized, crossover design, patients were randomly allocated to receive VIP (8 pmol/kg/min) or placebo (sterile saline) over 2 hours on 2 different study days that were separated by at least 2 weeks. All participants arrived headache free at the clinic on each study day. The experiment was postponed if the patient had experienced any kind of headache or used painkillers 48 hours before the start of the experiment. The intake of coffee, tea, cocoa, alcohol, or tobacco was not allowed for at least 8 hours before the start of the study. All procedures were performed in a quiet room with a temperature of 25 °C. The patient was placed in the supine position and 2 venous catheters were placed into the left and right forearms for experimental infusion and blood sampling. The patient rested for 30 minutes before baseline measurements were performed. Headache intensity–associated symptoms including nausea, photophobia and phonophobia, adverse events (AEs), and vital signs were recorded 10 minutes before baseline (T_-10_), at baseline (T_0_) and every 10 minutes after the start of the infusion, until 3 hours and 20 minutes (T_200_). Diameter of the STA was measured at T_0_, T_10_, T_30_, and every 30 minutes until T_180_. Cranial autonomic parasympathetic symptoms (CAPS) were recorded at baseline, infusion, and postinfusion time points.^23^ On completing the experimental procedure, patients were discharged and asked to complete a headache diary every hour for 12 hours after infusion of either VIP or placebo. The diary included headache characteristics, associated symptoms, intake of any rescue medication, adverse events, and premonitory symptoms.

### Randomization

Medical staff not involved in the study performed nonblocked randomization and preparation of study medications. The random allocation sequence was generated through a computerized random number generator. Allocation concealment was obtained using sequentially numbered, opaque, sealed envelopes. The envelopes received numbers in advance and were opened sequentially. The 2 solutions (VIP and placebo) looked identical and were administered using a time and volume-controlled infusion pump. The randomization code remained in the hospital during the study and was not available to the investigating physicians until the study was complete and data was analyzed. The randomization code was stored in a light-sealed envelope only to be broken in case of need for patient safety. There was an envelope for each patient to ensure the overall randomization code was not revealed in case any envelope was opened.

### Headache Recording and Provoked Migraine Attacks

Headache intensity was rated by a numerical rating scale verbally declared from 0 to 10, where 0 represented no pain; 1 was a very mild headache, including a feeling of pressing or throbbing; 5 was a moderate headache; and 10 was the worst possible headache. Experimentally provoked migraine is not spontaneous and, therefore, cannot fulfill strict ICHD-3 criteria for migraine without aura. Therefore, the following criteria were used.^24^

Provoked migraine attacks must fulfill either of 2 criteria. First, it must be a headache with at least 2 of the following clinical features: unilateral location, pulsating quality, moderate to severe pain intensity, and aggravation or avoidance of routine physical activity. Headache must also be accompanied by at least 1 of the following symptoms: nausea/vomiting or photo- and phonophobia. Second, it must mimic the patient’s usual migraine attack and be managed with a rescue medication.

### Hemodynamic Parameters

The diameter of the frontal branch of the STA was measured by a high-resolution ultrasonographic unit.^19^ Blood pressure and heart rate (HR) were measured using an autoinflatable cuff. Blood pressure was registered as mean arterial pressure (MAP). An electrocardiogram (ECG) was recorded at baseline and continuous ECG monitoring was also performed on each study day.

### Statistical Analysis

Mean (range), median (interquartile range [IQR]), and percentages were used for the description of all data. Calculation of sample size was based on the difference between 2 groups reporting migraine-like attacks after VIP and placebo at 5% significance with 80% power. We assumed that 60% of patients would report a migraine attack only after VIP and 20% would report an attack only after the placebo. We estimated that 20 patients should be included.^25^ This assumption was based on previous studies^10,11,26^ performed in patients with migraine after the administration of migraine-inducing peptides (ie, calcitonin gene-related peptide, PACAP27, and PACAP38).

The primary end point was the difference in the incidence of migraine attacks during the entire observational period (T_0_-T_12h_) comparing VIP with placebo. Secondary end points were (1) difference in area under the curve (AUC) for headache intensity scores during hospitalization (T_0_-T_12h_); (2) difference in AUC for the diameter of STA during hospitalization (T_0_-T_180min_); (3) difference in AUC for MAP during hospitalization (T_0_-T_200min_); and (4) difference in AUC for HR during hospitalization (T_0_-T_200_ min), comparing VIP with placebo. All other end points were considered exploratory.

According to the trapezium rule,^27^ we calculated AUC to obtain summary measures and to analyze differences between VIP and placebo. All statistical analyses were conducted between paired samples (eg, within participants). Baseline differences, as well as AUC values for headache scores, STA diameter (mm), MAP (mm Hg), and HR beats per minute were compared by a paired 2-way *t* test or Wilcoxon matched-pairs signed rank test, according to the normal distribution of data. Period and carryover effects for all baseline variables were tested with Mann-Whitney test and independent *t* test. Incidence of migraine attacks, head pain, associated symptoms, and adverse events was tested with McNemar test. Statistical analysis and graphs were performed using R version 4.0.2 (R Project for Statistical Computing) and Prism 8.3.0 (GraphPad). Level of significance at 5% (*P* < .05, 2-tailed) was accepted for all tests. Data collection was performed between May 2020 and September 2020. Data analysis was performed in October 2020.

## Results

**Figure 1.**
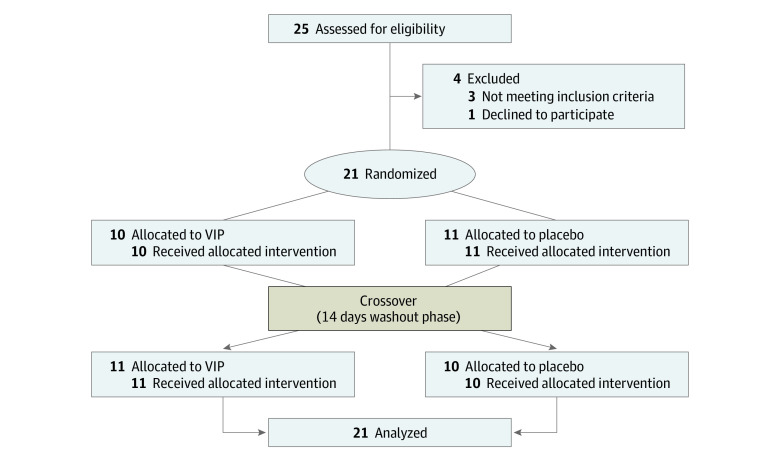
Flowchart of the Study VIP indicates vasoactive intestinal polypeptide.

A total of 21 patients were recruited and completed the study (17 [81%] women; 4 [19%] men; mean [range] age, 25.9 [19-40] years; mean [range] weight, 69.8 kg [52-89 kg]) (Figure 1). The frequency of baseline migraine attacks ranged from 1 to 6 attacks per month. Associated symptoms during spontaneous attacks were photophobia (18 [86%]), phonophobia (16 [76%]), nausea (15 [71%]), vomiting (5 [24%]), nasal congestion (2 [10%]), tearing (2 [10%]), facial redness (1 [5%]), and sensation of fullness in the ear (1 [5%]). There was no carryover or period effect for values of headache, diameter of STA, heart rate, and MAP.

### Provoked Migraine and Headache

**Figure 2.**
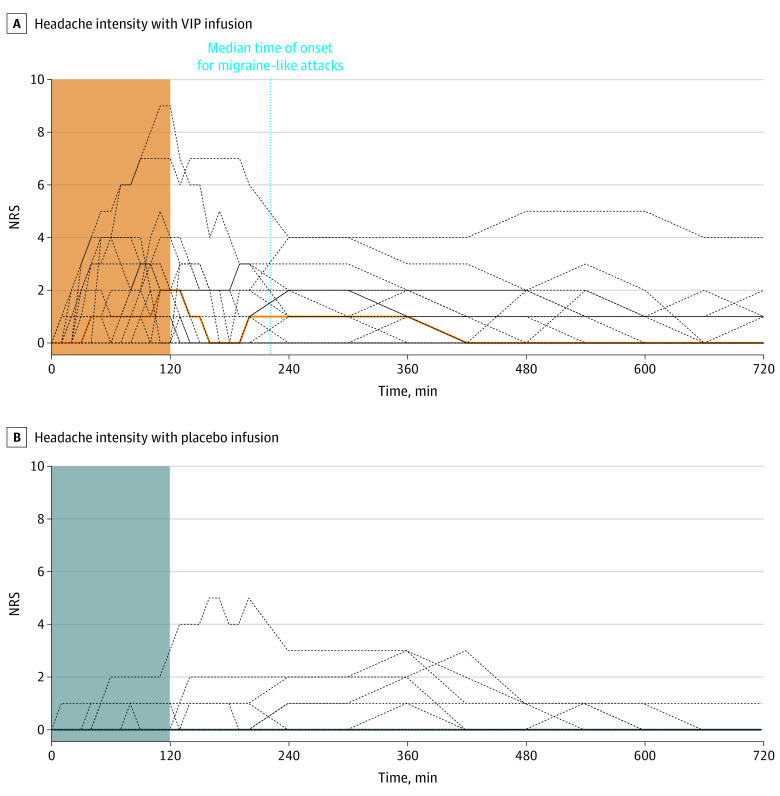
Headache Intensity on Vasoactive Intestinal Polypeptide and Placebo Days in 21 Patients With Migraine The abscissa refers to the time (min). (A) Median (thick, orange line) and individual (dotted lines) headache intensity on an NRS. The orange area represents the 2-hour infusion of VIP. The AUC for headache intensity was significantly greater after VIP compared with placebo (AUC_0-12h_, *P* = .003). The median time of onset for migraine-like attacks was 1 h 40 min (IQR, 1 h to 1 h 50 min). (B) Median (thick, black line) and individual (dotted lines) headache intensity on an NRS. The light blue area represents the 2-hour infusion of placebo. NRS indicates numerical rating scale.

**Figure 3.**
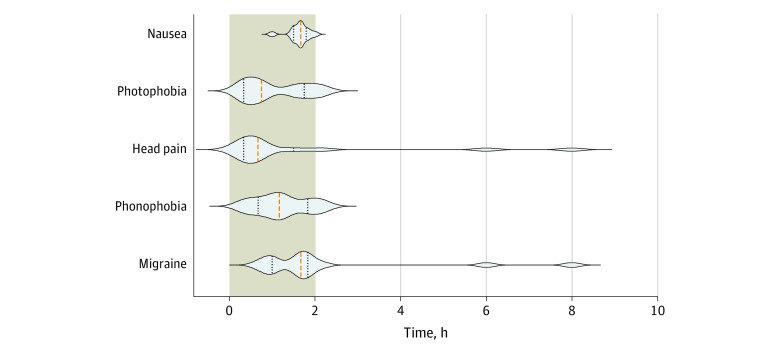
Violin Plot of the Time of Appearance of Head Pain, Nausea, Migraine-Like Criteria, Phonophobia, and Photophobia The orange dashed line represents the median, while the black dotted lines are the interquartile range. The light brown area represents the 2-hour infusion of vasoactive intestinal polypeptide. The width of the violin plot indicates the number of participants experiencing the symptom.

Of the 21 patients, 15 patients (71%; 95% CI, 48%-89%) developed migraine attacks after taking VIP, compared with 1 patient (5%; 95% CI, 0%-24%) after taking the placebo (*P* < .001). Patients reported that the induced attacks mimicked spontaneous migraine attacks in all cases. Median time to onset of migraine-like attacks was 1 hour 40 minutes (IQR, 1 hour to 1 hour 50 minutes). Headache localization was mainly in the frontal (14 [67%]) and temporal (10 [47%]) regions. Headache incidence over the entire 12-hour observational period was greater after VIP (19 [90%]) compared with placebo (7 [33%]; *P* = .02). AUC for headache intensity was greater after VIP compared with placebo (AUC_0-12h_, *P* = .003) (Figure 2). After VIP more patients reported nausea and photophobia but not phonophobia compared with the placebo (nausea: 18 [86%] vs 1 [5%]; *P* < .001; photophobia: 12 [57%] vs 0; *P* = .02; phonophobia: 9 [43%] vs 0; *P* = .08) (Figure 3). The median peak headache intensity was 3 (IQR, 2-5), and median time to peak headache occurred 1 hour and 50 minutes (IQR, 1 hour to 3 hours 10 minutes) after administration of VIP, which corresponded with the onset of migraine attacks. The median CAPS scores were higher during the VIP infusion (2 [IQR, 1.5-3]) compared with placebo (0; [IQR, 0-0]). Individual characteristics of headache, headache localization, and associated symptoms are reported in Table 1 and eTable 1 and eFigure 1 in Supplement 2.

**Table 1.** Clinical Characteristics of Headache and Related Associated Symptoms in Migraine Patients After Vasoactive Intestinal Polypeptide and Placebo (0-12 Hours Observational Period)

Patient No.	Peak headache (duration of headache), h	Headache characteristics							Associated symptoms			Mimics usual migraine	Onset of migraine-like attack, h^b^	Treatment (time)/efficacy^c^
		Localization	Intensity		Quality		Aggravated by cough or movement^a^		Nausea	Photophobia	Phonophobia			
**1**														
VIP	None	NA		NA		NA		NA	NA	NA	NA	NA	NA	NA
Placebo	None	NA		NA		NA		NA	NA	NA	NA	NA	NA	NA
**2**														
VIP	9 (4)	Bilateral		2		Pressing		Yes	Yes	Yes	Yes	Yes	NA	None
Placebo	None	NA		NA		NA		NA	NA	NA	NA	NA	NA	NA
**3**														
VIP	1.8 (11.7)	Bilateral		9		Pressing		Yes	Yes	Yes	Yes	Yes	1	Treo^d^ 1100 mg (2 h)/Yes
Placebo	None	NA		NA		NA		NA	NA	NA	NA	NA	NA	NA
**4**														
VIP	1 (1.8)	Unilateral		4		Throbbing		Yes	Yes	No	No	Yes	1.8	None
Placebo	None	NA		NA		NA		NA	NA	NA	NA	NA	NA	NA
**5**														
VIP	None	NA		NA		NA		NA	NA	NA	NA	NA	NA	NA
Placebo	None	NA		NA		NA		NA	NA	NA	NA	NA	NA	NA
**6**														
VIP	1.8 (1.7)	Bilateral		4		Pressing		Yes	Yes	Yes	No	Yes	2	None
Placebo	None	NA		NA		NA		NA	NA	NA	NA	NA	NA	NA
**7**														
VIP	1.8 (8)	Bilateral		2		Pressing		Yes	No	No	No	No	NA	None
Placebo	None	NA		NA		NA		NA	NA	NA	NA	NA	NA	NA
**8**														
VIP	8 (2)	Bilateral		2		Pressing		Yes	No	No	No	Yes	8	Sumatriptan 50 mg (8 h)/Yes
Placebo	0.2 (2)	Bilateral		1		Pressing		No	No	No	No	No	NA	None
**9**														
VIP	2.2 (0.7)	Unilateral		3		Pressing		Yes	Yes	Yes	Yes	Yes	2.2	None
Placebo	None	NA		NA		NA		NA	NA	NA	NA	NA	NA	NA
**10**														
VIP	1.5 (1.8)	Bilateral		3		Throbbing		Yes	Yes	Yes	No	Yes	1.8	None
Placebo	2.7 (7.5)	Unilateral		5		Pressing		Yes	Yes	No	No	Yes	1.5	None
**11**														
VIP	3.2 (10.5)	Unilateral		3		Throbbing		No	Yes	No	No	Yes	1.8	None
Placebo	0.8 (1.2)	Unilateral		1		Pressing		No	No	No	No	No	NA	None
**12**														
VIP	0.7 (0.8)	Unilateral		1		Throbbing		Yes	Yes	No	No	Yes	1.5	None
Placebo	None	NA		NA		NA		NA	NA	NA	NA	NA	NA	NA
**13**														
VIP	6 (2)	Unilateral		1		Throbbing		No	Yes	No	No	Yes	6	None
Placebo	7 (6)	Bilateral		3		Pressing		Yes	No	No	No	No	NA	Paracetamol 1 g (7 h)/Yes
**14**														
VIP	0.7 (7.7)	Bilateral		3		Throbbing		Yes	Yes	Yes	Yes	Yes	1	None
Placebo	None	NA		NA		NA		NA	NA	NA	NA	NA	NA	NA
**15**														
VIP	1.5 (12.0)	Bilateral		7		Pressing		Yes	Yes	Yes	Yes	Yes	1.5	Ibuprofen 800 mg (2 h)/Yes
Placebo	None	NA		NA		NA		NA	NA	NA	NA	NA	NA	NA
**16**														
VIP	8.0 (11.2)	Unilateral		5		Pressing		Yes	Yes	Yes	Yes	Yes	2.2	Rizatriptan 10 mg (8 h)/ Yes
Placebo	2.3 (5.2)	Bilateral		2		Pressing		No	No	No	No	No	NA	None
**17**														
VIP	0.3 (1.2)	Bilateral		1		Throbbing		Yes	Yes	Yes	Yes	Yes	1.7	None
Placebo	None	NA		NA		NA		NA	NA	NA	NA	NA	NA	NA
**18**														
VIP	0.3 (1.7)	Bilateral		2		Pressing		No	Yes	No	No	No	NA	None
Placebo	2.3 (0.8)	Bilateral		1		Pressing		No	No	No	No	No	NA	None
**19**														
VIP	1 (5.7)	Unilateral		4		Pressing		No	Yes	Yes	Yes	Yes	1	Zolmitriptan 5 mg (3 h)/Yes
Placebo	None	NA		NA		NA		NA	NA	NA	NA	NA	NA	NA
**20**														
VIP	1.3 (2.2)	Bilateral		2		Pressing		Yes	Yes	Yes	Yes	Yes	NA	None
Placebo	None	NA		NA		NA		NA	NA	NA	NA	NA	NA	NA
**21**														
VIP	1.8 (11.7)	Bilateral		5		Throbbing		Yes	Yes	Yes	Yes	Yes	1.8	Ibuprofen 400 mg (2 h)/Yes
Placebo	6 (6.8)	Bilateral		3		Pressing		No	No	No	No	No	NA	None

Abbreviations: NA, not applicable; VIP, vasoactive intestinal polypeptide.

^a^ Aggravated by cough during in-hospital phase or by movement during out-hospital phase.

^b^ Migraine-like attacks are defined according to the criteria described in the Methods section.

^c^ Pain freedom or pain relief (≥50% decrease of intensity) within 2 hours.

^d^ Combination of acetylsalicylic acid (500 mg) and caffeine (50 mg).

### Hemodynamic Variables

During the in-hospital phase, we found an increase of the STA diameter (AUC_0-180min_, *P* < .001) and heart rate (AUC_0-200min_, *P* < .001) after VIP compared with placebo. No difference was found regarding MAP (AUC_0-200min_, *P* = .73) between VIP and placebo. Detailed graphs are reported in eFigure 2 in Supplement 2.

### Adverse Events

The most common adverse events were flushing, warm sensations, heart palpitations, and cold sensations (flushing: 20 [95%] during VIP vs 3 [14%] during placebo; *P* < .001; warm sensations: 20 [95%] during VIP vs 2 [10%] during placebo; *P* < .001; heart palpitations: 17 [81%] during VIP vs 1 [5%] during placebo; *P* < .001; cold sensations: 12 [57%] during VIP vs 0 during placebo; *P* = .02). Warm sensations were mostly observed during the infusion period, while cold sensations were mainly observed in the postinfusion period. Other reported adverse events were abdominal discomfort, back pain, tiredness, and diarrhea. Adverse events are reported in eTable 2 in Supplement 2.

## Discussion

Our findings suggest that a 2-hour infusion of VIP induced migraine attacks in patients with migraine without aura. The induced migraine was associated with a prolonged dilation of STA and mimicked spontaneous migraine attacks, including localization and associated symptoms.

### VIP-Induced Migraine Attacks

In the present study, we found VIP had a 71% migraine induction rate. Applying the same criteria for migraine-induced attacks, VIP induced no attacks in 1 study^15^ and 4 attacks (18%) in another study^10^ conducted in patients with migraine without aura. Of note, the study reporting VIP-induced migraine attacks in 4 patients was not placebo-controlled, and findings might be consistent with the nocebo effect described in human models of migraine.^28^

In our study, the migraine induction rate of VIP is similar to the induction potential earlier reported after 20-minute infusion of PACAP38 (58%-73%).^9,10^ The median time to migraine onset (1 hour 40 minutes) was shorter after a 2-hour VIP infusion compared with earlier reported migraine inductions with PACAP27 (3 hours) and PACAP38 (4 hours 15 minutes)^10,11^ The incidence of associated symptoms, such as nausea (93%), photophobia (67%) and phonophobia (53%) was comparable with previously reported PACAP-induced migraine (nausea: 55%-72%; photophobia: 64%; phonophobia: 48%-55%).^9,10,11^ The high incidence of nausea, photophobia, and phonophobia in patient with migraine, compared with a previous study in healthy volunteers, suggests that these symptoms are not merely dose-related adverse events. In healthy volunteers, 2-hour infusion of VIP induced nausea and photophobia only in 33% and 8% of participants, respectively.^19^ None of the healthy volunteers reported phonophobia.^19^ At the same time, the dose-related side effects, such as flushing, warm sensations and heart palpitations, were very similar between the 2 study populations (Table 2).

**Table 2.** Migraine Characteristics and Physiological Effects in Participants Undergoing Randomized, Double-Blind, Placebo-Controlled, Crossover Trials With 2-Hour Intravenous Infusion of VIP

Incidence	No. (%)	
	Patients with migraine (n = 21)	Healthy volunteers (n = 12)^a^
Migraine attack	15 (71)	3 (25)
Nausea	18 (86)	4 (33)
Photophobia	12 (57)	1 (8)
Phonophobia	9 (43)	0
Flushing	20 (95)	12 (100)
Warm sensation	20 (95)	12 (100)
Heart palpitation	17 (81)	12 (100)

Abbreviation: VIP, vasoactive intestinal polypeptide.

^a^ Data were previously published in Pellesi et al.^19^

### Potential Mechanisms

The major difference between our study and previous studies of migraine provocation is the duration of infusion. We previously demonstrated that a prolonged VIP infusion caused sustained STA dilation in healthy volunteers. In this study, our goal was to induce a similar long-term dilation in patients with migraine and to observe whether this would lead to migraine. With our results of 71% migraine induction and prolonged STA dilation, the question arises whether we can explain VIP-induced migraine attacks by prolonged extracranial artery dilation. Early observations pointed out the association of distension of cranial arteries and head pain.^29,30^ Vasoactive peptides activate receptors expressed on smooth muscle cells and upregulate intracellular cAMP, initiating a signaling cascade that results in opening of potassium channels and vasodilation.^31^ Dilation of cranial arteries induced by VIP and PACAPs is predominantly mediated by vasoactive intestinal polypeptide receptor 1 (VPAC_1_) and vasoactive intestinal polypeptide receptor 2 (VPAC_2_) receptors.^32,33,34,35^ A 2-hour VIP infusion and prolonged stimulation of the VPAC_1_ and VPAC_2_ receptors may cause an increase in open probability of potassium channels and a substantial potassium ions efflux.^36,37^ Accumulation of extracellular positively charged ions would result in depolarization and thus activation of the trigeminal pain pathway.^38,39,40^ This basic mechanism may explain the provoked migraine after a 2-hour VIP infusion. In support, direct activation of adenosine triphosphate-sensitive potassium (K_ATP_) channels or big-conductance calcium-activated potassium (BK_Ca_) channels induced prolonged dilation of cranial arteries and triggered migraine attacks in more than 90% of all participants.^41,42^ Collectively, the present study implicates VPAC_1_ and VPAC_2_ receptors in the pathogenesis of migraine, and selective antagonists might be of future interest for the treatment of migraine.

The biological effects of VIP and PACAP are mediated via VPAC_1_ and VPAC_2_ and pituitary adenylate cyclase-activating polypeptide type I receptor (PAC_1_).^43^ Both peptides and its receptors are expressed in smooth muscle cells^44,45,46^ and neurons and glial cells of the trigeminal and sphenopalatine ganglion.^47,48^ Furthermore, PAC_1_ receptors are expressed in trigeminal and sphenopalatine ganglion neurons as well as the spinal trigeminal nucleus,^46,47,48^ whereas their presence in the cranial vasculature is debatable.^44,46,49,50^ Of note, maxadilan, a selective PAC_1_ receptor agonist, did not show a relaxant effect in rat intracranial and extracranial arteries.^32^ In rats, the nociceptive activity of trigeminocervical neurons was reversed by the administration of a PAC_1_ antagonist.^35,51^ To date, the difference between VIP and PACAP in migraine induction has been mainly attributed to a low affinity of PAC_1_ receptors for VIP.^43^ However, a recent study has questioned the role of this receptor in initiating migraine. In a randomized clinical trial, a monoclonal antibody targeting the PAC_1_ receptor (AMG 301) failed in migraine prevention.^52^ The data in the current study on VIP-induced migraine provide alternatives to the role of PAC_1_ receptor in migraine induction. Because VIP has a very low affinity for the PAC_1_ receptor, it is unlikely that VIP-induced migraine was mediated via this receptor.

### Limitations

This study has limitations. Different factors might have affected the incidence of migraine-like attacks. However, the consolidated experimental conditions make results robust. In the placebo arm, the incidence of migraine attacks (5%) was comparable with previously reported studies with migraine-inducing substances.^28^ The sample size of the study was calculated beforehand, based on previous studies with PACAP38 and CGRP.^53^ As with all new results, our data demands confirmation in different cohorts.

## Conclusions

Our findings revisit the role of VIP in migraine pathogenesis and suggest that a prolonged arterial dilation might be involved in the initiation of migraine attacks. A possible mechanism relates to the activation of VIP receptors on smooth muscle cells, the increase in intracellular cAMP and the activation of ion channels, and the consequent activation of perivascular nerve fibers. We suggest that selective antagonists of VIP and its receptors could be potential targets for novel drugs for migraine.

## Notes

Supplement 1. Trial Protocol

Supplement 2. **eTable 1.** Headache-Associated Symptoms Recorded and Reported During Infusion and Postinfusion Periods **eTable 2.** Most Common Adverse Events Recorded During the Hospitalization Period **eFigure 1.** Localization of Headache During the Migraine-Like Attacks Induced by 2-Hour Infusion of VIP Compared With Spontaneous Migraine **eFigure 2.** Haemodynamic Effects of VIP

Supplement 3. Data Sharing Statement
