# Supplementary material for: Effect of Vasoactive Intestinal Polypeptide on Development of Migraine Headaches: A Randomized Clinical Trial
Source: JAMA Netw Open. 2021 Aug 6;4(8):e2118543. doi: 10.1001/jamanetworkopen.2021.18543 (PMC8346940; doi:10.1001/jamanetworkopen.2021.18543)
Supplement: Supplement 1. — Trial Protocol [file jamanetwopen-e2118543-s001.pdf]

01/04/2020

PROTOCOL

The effects of a long-lasting infusion of vasoactive intestinal peptide (VIP) on headache, cranial hemodynamic and autonomic symptoms in episodic migraine patients without aura

Version 1.2

Mohammad Al-Mahdi Al-Karagholi, MD, Ph.D. student

Lanfranco Pellesi, MD

Faisal Mohammad Amin, MD, Ph.D.

Henrik Larsson, Prof.

Messoud Ashina, DrMSc, Prof.

Ansvarlig læge og forsøgssted

Mohammad Al-Mahdi Al-Karagholi, MD

Dansk Hovedpinecenter, Neurologisk klinik

Rigshospitalet-Glostrup

Valdemar Hansens vej 5 indgang 1 A

2600 Glostrup

Tlf.: 31 19 16 47

E-mail: Mahdi.alkaragholi@gmail.com

Indholdsfortegnelse

1. Title, Place and Purpose…………3
4. Background...…………3
5. Hypotheses…………4
6. Description of VIP…………4
6.1 Pharmacokinetics and metabolism…………...4
6.2 Pharmacodynamics…………...4
6.3 Safety profile and adverse events…………5
6.4 The dose and the preparation…………6
7. The Experiment…………6
7.1 Participants…………...6
7.2 Recruitment…………..6
7.3 Screening visit…………......6
7.4 Inclusion criteria…………...7
7.5 Exclusion criteria…………..7
7.6 Design…………...7
7.7 Experimental days...…………..9
8. Methods…………9
8.1 Headache registration…………...9
8.2 Hemodynamic parameters…………..10
8.3 Kinetic oscillation stimulation (KOS)...…………10
8.4 Assessment of lacrimation…………..10
8.5 Blood samples…………10
8.6 Number of participants…………...11
8.7 Calculations and statistical methods…………...11
9. Risk assessment…………...11
10. Unintended events….…………...12
11. Time Frame…………..12
12. Publication…………....12
13. Economy…………..13
14. Insurance…………..13
15. Ethics…………...14
16. Responsible department…………15
17. Study investigators…………..15
References…………...…………...16

1. Title

The effects of a long-lasting infusion of vasoactive intestinal peptide (VIP) on headache, cranial hemodynamic and autonomic symptoms in episodic migraine patients without aura.

2. Place

Dansk Hovedpinecenter, Neurologisk klinik, Rigshospitalet-Glostrup, Valdemar Hansens vej 5 indgang 1A, 2600 Glostrup.

3. Purpose

To use a long-lasting infusion of VIP and placebo in episodic migraine patients without aura to:

- Investigate the occurrence of headache.
- Investigate the cranial hemodynamic.
- Investigate the cranial autonomic symptoms.

4. Background

Vasoactive intestinal peptide (VIP) is a peptide of 28 amino acid residues that belongs to the glucagon/secretin superfamily of peptides. It is produced in different regions of the nervous system, including the brain, trigeminovascular system and several autonomic nerves (1). Once released from neurons, it acts on vasoactive intestinal peptide receptor 1 (VPAC1), vasoactive intestinal peptide receptor 2 (VPAC2) and pituitary adenylate cyclase-activating polypeptide type I receptor (PAC1), by mediating smooth muscle relaxation, vasodilation and water secretion (2). Along with other neuropeptides, such as calcitonin gene-related peptide (CGRP) and pituitary adenylate cyclase-activating polypeptide (PACAP), it is released from the trigeminal afferents and exerts a strong vasodilating activity on the cranial vasculature, sharing the activation of adenylate cyclase (3). Especially, it shares 70% structure with PACAP and acts on the same receptors. But, unlike it, VIP cannot induce a long-lasting vasodilation and has a modest capability to induce migraine attacks (4,5,6,7). A two-hour infusion of VIP showed a long-lasting vasodilatation of the cerebral vessels in healthy volunteers (data not published), but whether it may induce migraine-like attacks in migraine patients, as a twenty-minute infusion of PACAP, is unknown.

.

5. Hypothesis

Long-lasting infusion of VIP provokes migraine-like attacks, causes a prolonged cranial vasodilation and autonomic symptoms.

6. Description of VIP

VIP is a 28-amino acids peptide with a huge structural similarity with other vasodilating peptides, such as peptide histidine methionine (PHM) and PACAP. In humans, VIP is found in the autonomic nervous system (11) and also around intra- and extracerebral blood vessels (12). To date, there are different receptors for VIP, the most popular are VPAC1 and VPAC2 (13).

6.1 Pharmacokinetics and metabolism

A pharmacokinetic study with 4 healthy participants was performed, with an intravenous VIP infusion over 30 min, with doses of 0.6, 1.3 and 3.3 pmol / kg / min. It increased the plasma concentration with the two highest doses to 154 pmol / liter and 351 pmol / liter, respectively. The steady-state was obtained after 15 min and the elimination followed first order kinetics with estimated half-time, T½, to 1 min. The elimination rate was calculated to 9 ml/kg/min and the distribution volume was 13 ml/kg (14).

6.2 Pharmacodynamics

The effect of exogenous VIP is described in several human studies (6,15). VIP has well-documented effects in the brain, the digestive tract, the airways, the cardiovascular system, the immune system, the endocrine gland and the reproductive organs (16,17).

6.2.1 Effect on blood circulation and lungs

VIP has a direct positive inotropic and chronotropic effect on the heart. However, VIP reduces the vascular resistance and thus decreases the blood pressure (18). Frase et al. calculated a decrease in total peripheral resistance to 30% at a dose of 6.67 pmol / kg / min (18).
The effect on blood pressure and heart rate disappears within 1 hour after completion of infusion (19).
In addition, VIP was bronchodilator in asthma patients but not in healthy participants (20).

6.2.2 Effect on the digestive tract

In the Verner-Morrison syndrome, long-standing plasma levels of VIP are the main cause of aqueous diarrhea (21). In all human studies, plasma concentrations of VIP have been achieved at the same level as seen in the Verner-Morrison syndrome. In a diarrhea-inducing study with VIP infusion (6.67 pmol / kg / min) for 10 hours, all 5 participants had diarrhea after 4.3 hours, and it lasted for 4 hours after completion of infusion (22). In other studies, VIP infusion for more than 60 min. increased water excretion / decreased water absorption from the small intestine (21,23). In all other studies where the infusion time of VIP was shorter than 60 min. no diarrhea (6,7,15) was reported.

VIP infusion for 2 hours increases bile secretion, which was normalized 2 hours after completion of infusion (24).

6.2.3 Effect on cerebral blood vessels

No changes were found in either global or regional cerebral blood flow after VIP infusion in healthy participants (6). Furthermore, decreases in velocity in middle cerebral artery (MCA) was reported in migraine patients following VIP infusion (15).

6.3 Safety profile and adverse events

A 2-hour infusion of VIP was recently conducted in healthy volunteers by our team, without highlighting serious safety issues (data not published). The most common adverse events related to VIP infusion are flushing and heat sensation, and they disappear 15 min after the completion of infusion (6,7,15). Changes in blood pressure and heart rate were dose-dependent and were often normalized within 1 hour after the infusion (6,7,15). Headache was also reported after VIP infusion from different studies (6,7,15,21). Diarrhea was only induced after a long-lasting infusion of VIP (22).

6.4 The dose and the preparation

Intravenous infusion of VIP up to 15 pmol / kg / min has been given in human studies, for 15-30 min (14,19,25). A 2-hour infusion of VIP was recently conducted in healthy volunteers by our team (data not published). This dosage did not result in any further influence on hemodynamic parameters than expected, e.g. decrease in blood pressure and increase in heart rate. In addition, no serious side effects were observed. Significant changes in hemodynamic parameters are seen at doses from 2 pmol / kg / min and are dose-dependent (14,19,25). Other human studies have administered VIP infusions up to 7 pmol / kg / min for 10 hours (22,26). Additionally, VIP might cause flushing and diarrhea, but no serious or unacceptable side effects are envisaged. In this study, we aim to use 8 pmol / kg / min VIP over 120 minutes in episodic

migraine patients without aura. VIP has a purity of at least 98% verified by high-performance liquid chromatography (HPLC). VIP will be sterilized and packed at Central Pharmacy in Herlev.

7. The experiment

7.1 Participants

We will recruit 20 episodic migraine patients without aura.

7.2 Recruitment

Episodic migraine patients without aura will be contacted among those who are afferent to the Dansk Hovedpinecenter. Participants form previous studies who have expressed their interest in future clinical trials will be contacted by telephone. New participants will be recruited by alerts on closed groups in social networks (Facebook) (see Appendix F). All interested participants will receive a written information material and will be invited for a screening visit.

7.3 Screening visit

Before the screening visit, the participants will receive approved participant information and the "Before You Decide" folder published by Danish Authority of the Ministry of Health and Prevention. The attending physician or a person to whom the responsibility is delegated will inform the participant thoroughly at the subsequent meeting about the purpose and possible risks of the study. By telephoning the participant we will plan a screening visit at Valdemar Hansens Vej 5 indgang 1 A, Rigshospitalet-Glostrup Hospital. The screening visit takes place in an undisturbed physical environment. At the screening visit the participant will again be given written participant information and will also be informed orally about the purpose, course and possible risks in the study.

A medical chart will be recorded, and a general medical examination will be conducted as well. ECG will also be taken. This will be done to uncover any underlying risk factors and to ensure that the participants meet the inclusion criteria and do not meet the exclusion criteria. The participants will also be informed of the right to withdraw from the study without having to argue for this without affecting any future treatment.

7.4 Inclusion criteria

- Diagnosis of migraine without aura as according to the International Classification (27).
- Frequency of migraine attacks between one and six attacks within 8 weeks.
- Age: 18-40 years.
- Weight: 50-90 kg.

7.5 Exclusion criteria

• Any other type of headache (including > 2 days of tension-type headache per month), according to the International Classification (27).

• Headache less than 48 hours before the start of the experiment.

• Fertile women that are not using contraception. Contraception includes either IUD, birth control pills, surgical sterilization of the woman or depot progesterone. Fertile women do not include hysterectomies women or women who are postmenopausal for at least 2 years.

• Pregnant or breastfeeding women.

• Daily intake of any medicine other than oral contraception. A three-month wash out is required for previous daily intake of medications.

• Clinical signs of:

- Hypertension (systolic blood pressure > 150 mmHg and / or diastolic blood pressure > 100 mmHg)
- Hypotension (systolic blood pressure < 90 mm Hg and / or diastolic blood pressure < 50 mmHg)

• Cardiovascular disease of all kinds, including cerebrovascular disease.

• Anamnestic or clinical signs of mental illness, abuse or smoking.

• Anamnestic or clinical signs of diseases of any kind considered by the investigating physician relevant for participation in this study.

• Contraindications to MRI scanning (including metal in the body or claustrophobia).

7.6 Design

Pilot study

The first part consists of a pilot study. In three episodic migraine patients without aura, the effects on the cerebral arteries will be evaluated by MRI. Every participant will be placed in the MRI scanner and the following will be done:

1. Baseline measurement of diameter, circumference and blood flow of STA, MMA and MCA, as well as blood pressure, pulse, headache and accompanying symptoms
2. MR measurements are repeated 10, 50, 100, 140 and 180 minutes after the start of the infusion
3. From the start of the infusion, blood pressure, heart rate, headache response and accompanying symptoms will be recorder every ten minutes
4. After the administration and between MR measurements, the subject is removed from the scanner and placed in a bed or allowed to walk freely.

Main study

20 episodic migraine patients without aura of both genders will be included in the main study (see Figure 1). Participants will meet twice, with at least one week in between. The main study is a double blind, randomized, cross-over trial among placebo (sterile saline, non-active substance) and VIP. Intravenous infusion of VIP / placebo (saline) will be conducted over 120 minutes. Patients will be asked after each trial day to record headache strength, accompanying symptoms, and drug use for up to 24 hours post-infusion.

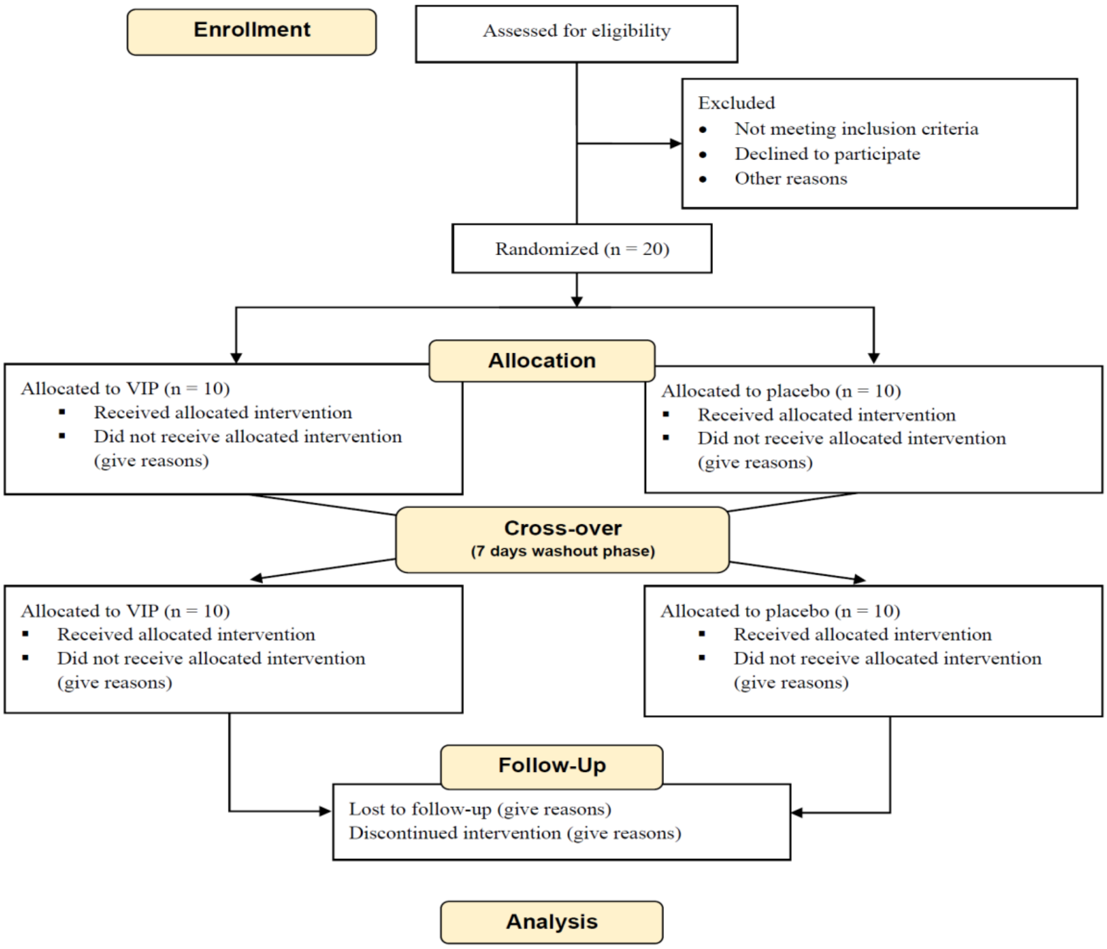

Figure 1. Timeline of the study

Measured parameters in the main study

We will measure the following:

a. Headache response, accompanying symptoms and adverse events.

b. Changes in vital parameters, i.e. mean arterial blood pressure (MAP) and heart rate (HR).

c. The diameter of STA will be evaluated by using Dermascan.
d. As local measures of autonomic activity, the Schirmer tearing test will be performed at the baseline, 30 minutes and one hour and 30 minutes after the start of the infusion. The last measurement will be conducted during a KOS treatment in the nasal cavity.
e. Cranial Autonomic Parasympathetic symptoms (CAPS) questionnaire (see Appendix G) will be performed at the baseline, during and after infusion.
f. Blood samples to measure serum VIP concentration.
Part a and b are registered at the baseline and every 10 minutes for the following 200 minutes (see Appendix D). The study of the STA with Dermascan will be performed in a timely manner, i.e. at the baseline and 10, 30, 60, 120, 150 and 180 minutes after the administration. Blood samples will be performed at baseline, 10, 20, 30 minutes after the start of the infusion and every 30 minutes until the discharge. On discharge, the headache and accompanying symptoms will be recorded at home (see Appendix E).

Randomization

Randomization is designed so that there are equal numbers of participants who receive active preparation on the first and second experimental days. The study investigator and other participants in the study do not have access to the randomization code. It is stored where the test is carried out in a light-sealed envelope and may be broken in cases where it is necessary for the safety of the participants. There will be an envelope for each subject so that the overall randomization code is not revealed in case an envelope is broken. The only one who has access to the randomization code is Professor Messoud Ashina.

7.7 Experimental days

The participants must not have headache at the experimental days and must be headache free for at least 48 hours before the start of the experiment. For 12 hours up to the start of the study, the subjects should not drink or eat coffee, tea, cocoa, cola or alcohol and should not smoke, as these substances affect the brain's blood flow and headache. Pregnancy test (urine HCG) will be performed for women with childbearing potential will. After arrival, baseline values of headache intensity are first performed using a 0-10 numerical Rating Scale (NRS) as well as heart rate and blood pressure. The participant meets 2 times (in addition to the screening visit) and receives infusion of VIP (8 pmol/kg/min for 120 min) or placebo (saline, non-active substance).
Before the start of the study, the participant is placed in a bed and an intravenous cannula is placed in the right or left elbow veins used for the infusion of VIP or placebo (sterile saline). On the other arm, an additional peripheral venous catheter will be inserted for the collection of blood samples. During the two hours of infusion, isotonic saltwater (500 ml) will be administered to prevent the hypotensive effects of VIP. There will be a doctor at the same room throughout the study. After 20 minutes of rest, baseline values are

noted for heart rate, blood pressure and hemodynamic parameters. After this, the infusion of VIP (8 pmol/kg/min) or placebo (sterile saline) will get started and will last for 120 min. Heart rate, blood pressure, hemodynamic parameters, and possible headache intensity and accompanying symptoms are recorded every 10 minutes during the infusion, and then upon discharge. The participant lies in bed throughout the experiment (0-4 hours). After this, the participant can go home if he / she feels ready for it.
The participant will be given a questionnaire (Appendix E) for home registration of any headaches and other symptoms. If the participant develops headache or migraine-like symptoms during the study, treatment will be offered in the form of 1000 mg of paracetamol, 400 mg ibuprofen or 6 mg subcutaneous sumatriptan. The patient is also given pain medication for any later needs at home.
The study includes a screening visit (1 hour) and two experimental days (about 4 hours each), a total duration of approximately 10 hours.

8. Methods

The purpose of the study is to investigate the occurrence of migraine-like attacks, changes in hemodynamic parameters and cranial autonomic symptoms after a long-lasting intravenous infusion of VIP compared with placebo (sterile saline). The study is randomized, double-blind, placebo-controlled and two-way crossover.

8.1 Headache registration

Headache is rated by a numerical rating scale (NRS). 0 represents no pain, 1 an "altered, pressing or throbbing but not really painful feeling", 5 moderate headache and 10 the worst possible headache. Headache, migraine characteristics and accompanying symptoms are classified according to IHS criteria (27). Other effects are recorded qualitatively using the form (Appendix D). Headache intensity and possibly accompanied symptoms are recorded qualitatively every 10 min, up to 200 minutes after the start of the infusion. After that, the participant is discharged with a headache questionnaire, to be completed at home (Appendix E). When the headache questionnaire is completed, it should be sent to the Danish Headache Center. The questionnaire also clarifies the clinical characteristics of any headache and accompanying symptoms.

8.2 Hemodynamic parameters

The diameter, circumference and blood flow of STA, MMA and MCA are measured by 3 Tesla MR-scanner (3T Philips Intera Achieva). Changes in diameter, circumference and blood flow will be analyzed by a specific software (LKEB MRA VesselWall Analysis). The diameter of STA is measured by Dermascan C, Cortex Technology, Hadsund, Denmark.
Pulse and blood pressure are measured automatically and intermittently with cuff (Protocol, Oregon, USA).

8.3 Kinetic oscillation stimulation (KOS)

KOS (Chordate Medical AB, Stockholm, Sweden) stimulation consisted of mechanical vibrations created using regular pressure oscillations at a frequency of 50 Hz. It is applied in a single nasal cavity, for 5 minutes, to activate the cranial autonomic pathways and to provoke lacrimation. A single use catheter will be coated with paraffin and inserted into the participants´ left nostril. To prevent the movement of the catheter, it will be secured with a headband holder. The catheter will be connected to a controller, allowing inflation and oscillation of the tip.

8.4 Assessment of lacrimation

Lacrimation as a quantifiable measure of autonomic activation will be recorded using Schirmer´s test II. After the administration of one drop (1 ml) Oxybuprocaine to both eyes, the anesthesia will be incubated for 5 minutes to ensure non-painful measurement of lacrimation. The sterile tear strips will be placed in the lower eyelid of both eyes and lacrimation will be recorded for a total time of 5 minutes. Three tests were applied, one at the baseline and two during the VIP long-lasting infusion.

8.5 Blood samples

Blood samples will be performed at baseline, 10, 20, 30 minutes after the start of the infusion and every 30 minutes until the discharge. Samples will be used to measure plasma concentrations of VIP, PACAP38 and CGRP. The purpose of the blood samples is to investigate the concentration changes of VIP and other related molecules that are related to VIP.

8.6 Number of participants

We assume that the number of migraine patients who will develop headache after placebo is 20%, and the number of patients who will develop headache after two-hour infusion of VIP is 60%. Significance level (α) is set to 5%. The strength (1-β) is set to 80%. Accordingly, the required number of participants is 20 (28).

8.7 Calculations and statistical methods

Primary end point is

a. Headache response between a long-lasting infusion of VIP and placebo

Secondary end points are

b. Changes in the diameter of superficial temporal artery (STA)
c. Changes in Schirmer´s test scores
d. Changes in vital parameters
e. Accompanying symptoms, CAPS questionnaire and adverse events

Data will be presented like an area under the concentration-time curve (AUC). Analysis will be performed with a paired, two-way *t test*, except headache scores, which will be tested with non-parametric test (Wilcoxon signed rank sum test). For the secondary endpoints, ANOVA with Tukey post-hoc correction will be used to

test the changing over time in Schirmer´s test scores, blood pressure and heart rate between the two experimental days.

9. Risk assessment

VIP is a vasoactive substance that has been used in various human studies. The frequent side effects reported are flushing and changes in blood pressure and hearts rate. In the recent study conducted in healthy volunteers, administering 2-hour infusion of VIP, no serious concerns raised on the risks associated with its administration. According to those results, we are planning to administer the same amount of VIP in migraine patients, thus examining the headache response.

VIP infusion will be stopped with significant effects of

1. Blood pressure:

a. Hypertension (systolic blood pressure > 170 mmHg and / or diastolic blood pressure > 120 mmHg).

b. Hypotension (systolic blood pressure < 80 mmHg and / or diastolic blood pressure < 50 mmHg).

2. Heart rate:

a. Accelerated cardiac action > 100 minutes, persistent for more than 5 minutes.

b. Slow cardiac action < 50 minutes, persistent for more than 5 minutes.

3. Unacceptable side effects, such as strong headaches.

4. Generally increased risk for unacceptable adverse events.

Intravenous cannula and assessment of vital parameters

Insertion of intravenous cannula may cause a subcutaneous hematoma that disappears after a few days and in very rare cases, infection. There are no known side effects, risks or significant discomfort associated with pulse and blood pressure recording.

Schirmer´s test

Schirmer´s test is a non-invasive examination that measures the production of tears. It lasts about 5 minutes and the preparation is minimal and simple. After instilling numbing drops, the medical doctor places the strip of paper inside the lower eyelids and the individual will keep their eyes closed for 5 minutes. Most people consider the test to be marginally irritating or uncomfortable. After the test, people should avoid wearing contact lenses for about 2 hours. Moreover, rubbing the eyes is not recommended for about 30 minutes. Besides these considerations, there are no other short- or long-term side effects to the procedure.

KOS stimulation

The parasympathetic stimulation is administered using a minimally invasive system. It consists of a controller and a single-use catheter, as well as a placebo module (CT100) and a headband (E100) from

Chordate Medical AB, Stockholm, Sweden. This method has already been used for 30 minutes in patients with migraine, without the occurrence of adverse events (29). In a larger group of patients with non-allergic rhinitis, the device was used for 14 minutes (30). In the latter study, several patients reported mild side effects, such as sneezing, repeated itchiness in and around the cartilaginous part of the nose and reduced sensitivity in the upper lip during treatment. All the symptoms normalized in the post-treatment.
In this study, the device will be used for five minutes, in order to stimulate the parasympathetic pathways from a nasal cavity. This approach is more similar to that already documented by other studies (31,32), where no adverse events are reported.

10. Unintended events
All adverse events (AEs), serious and non-serious, will be recorded in the case report form. Serious events which are presumed to be related to the drug, will be notified immediately to the Danish Medicinal Agency pursuant to the Medicines Act section § 89. Serious AEs also require individual expedited reporting to Novartis Chief Medical Office and Patient Safety. The investigator must also instruct each patient to report any new adverse event (beyond the observational period in the hospital) that the patient, or the patient's physician, believes might reasonably be related to study procedures. This information must be recorded in the investigator's source documents; if the AE meets the criteria of a serious AE, it must be reported to Novartis. Serious AEs must be reported to Novartis within 24 hours of the investigator learning of its occurrence/receiving follow-up information.

In this context, an unintended event is defined as any undesired event that is temporarily related to the administration of VIP/placebo, whether or not this accidental event is considered to be associated with VIP/placebo. If an unintended event occurs more than 14 days after the last administration of VIP/placebo, and there is no apparent causal link or association with VIP/placebo, this is not considered an unintended event.
In this context, a serious accidental incident means any medical case regardless of dose which

- results in death
- is life threatening
- involves hospitalization or extension of existing hospitalization
- results in persistent or significant disability / incapacity or
- is a congenital anomaly / malformation

If the participant develops headache or migraine-like symptoms during the study, treatment will be offered in the form of 1000 mg of paracetamol, 400 mg ibuprofen or 6 mg subcutaneous sumatriptan. The patient is also given pain medication for any later needs at home. All tests are performed in the presence of a medical

doctor with the possibility of calling for necessary assistance when needed. The department has access to O_2, resuscitation equipment, medicine and infusion fluids for the treatment of acute medical conditions. This protocol shall be submitted to the Scientific Ethics Committee for the Capital Region and the Data Inspectorate in accordance with applicable rules.

11. Time frame

The experiment is scheduled to take place between February 2020 and September 2020.

12. Publication

The paper will be sent to a peer reviewed international journal. Positive, negative, and inconclusive results will be published. The study is expected to result in a scientific paper with the following authors: Lanfranco Pellesi, Mohammad Al-Mahdi Al-Karagholi, Faisal Mohammad Amin, Henrik Larsson and Messoud Ashina.

13. Economy

Disability allowance for participants is 200 DKK per hour, approximately 10 x 200 kr. = 2000 kr. per participants (taxable). The researcher, Mohammad Al-Mahdi Al-Karagholi, PhD student at the Danish Headache Center is the contact person and the initiator of the study. We expect the project to cost approx. DKK 100,000, which goes towards preparing VIP, fees for patients and other costs including blood tests and ECG. The project is covered by grants received from Novartis. The remaining amount will be covered by The Headache Research Fund, Rigshospitalet-Glostrup, which includes funds under public audit.

The research project is planned and carried out by the experimental physicians. None of the doctors involved receives remuneration for conducting the trial. None of the investigators are financially linked to private companies, foundations etc. who have interests in the research project in question.

14. Insurance

If during the study or as a result of it any injury or complications for the study participants occurs, they will be covered by Rigshospitalet-Glostrup insurance.

15. Ethics

The study will be conducted according to the Helsinki Declaration of 1984, modified at the 59th World Congress in Seoul, South Korea 2008. The study must be approved by the Local Science Ethics Committee. Participants will only be included after full written and oral information and written acceptance. The study participants may withdraw from the trial at any time without justification and without it affecting future treatment.

Not serious adverse events are expected. The risk of serious adverse events is estimated to be very small. Should unexpected events such as allergic reactions occur, despite the anticipation, there is an action plan for observation and treatment of the participant.
This study will help to clarify the role of VIP in migraine disorder. This is of great interest in future research, as such knowledge is an important prerequisite for the development of better migraine medicine with fewer systemic side effects in the short and long term.
It is our opinion that the disadvantages, discomfort and risk of participants in this study are proportional to the significance of the expected results. Processing of personal data will be complied with in accordance with the law and data protection rules.
All data collected will be treated confidentially and only published in anonymized form. Raw data and randomization codes are stored in anonymized form under safe conditions for 15 years after the end of the study. If information from patient records is required, consent is obtained from the patient in accordance with section 43 of the Health Act. 1. The information will be used to screen the patient for the inclusion and exclusion criteria for the experiment.

16. Department responsible for the study

Neurological dept. N, Danish Headache Center, Valdemar Hansenvej 5 Indgang 1A, 2600 Glostrup

17. Study investigators

Mohammad Al-Mahdi Al-Karagholi, MD, Ph.D. student
Lanfranco Pellesi, MD
Faisal Mohammad Amin, MD, Ph.D.
Henrik Larsson, professor
Messoud Ashina, DrMSc, Prof.

The study will be performed, and participant information will be provided by Mohammad Al-Mahdi Al-Karagholi, or by specially trained staff under the supervision of the study medical doctors.

References

1. Gulbenkian S, Uddman R, Edvinsson L. Neuronal messengers in the human cerebral circulation. Peptides. 2001; 22(6): 995-1007.

2. Harmar AJ, Fahrenkrug J, Gozes I, Laburthe M, May V, Pisegna JR, Vaudry D, Vaudry H, Waschek JA, Said SI. Pharmacology and functions of receptors for vasoactive intestinal peptide and pituitary adenylate cyclase-activating polypeptide: IUPHAR Review 1. Br J Pharmacol. 2012; 166(1): 4–17.

3. Tajti J, Szok D, Majláth Z, Tuka B, Csáti A, Vécsei L. Migraine and neuropeptides. Neuropeptides. 2015; 52: 19-30.

4. Lassen LH, Haderslev PA, Jacobsen VB, Iversen HK, Sperling B, Olesen J. CGRP may play a causative role in migraine. Cephalalgia 2002; 22: 54–61.

5. Schytz HW, Birk S, Wienecke T, Kruuse C, Olesen J, Ashina M. PACAP38 induces migraine-like attacks in patients with migraine without aura. Brain. 2009; 132(1): 16-25.

6. Rahmann A, Wienecke T, Hansen JM, Fahrenkrug J, Olesen J, Ashina M. Vasoactive intestinal peptide causes marked cephalic vasodilation, but does not induce migraine. Cephalalgia 2008; 28: 226-236.

7. Amin FM, Hougaard A, Schytz HW, Asghar MS, Lundholm E, Parvaiz AI, et al. Investigation of the pathophysiological mechanisms of migraine attacks induced by pituitary adenylate cyclase-activating polypeptide-38. Brain 2014; 137: 779-794.

8. Goadsby PJ, Edvinsson L, Ekman R. Vasoactive peptide release in the extracerebral circulation of humans during migraine headache. Ann. Neurol 1990; 28: 183-187.

9. Riesco N, Cernuda-Morollón E, Martínez-Camblor P, Pérez-Alvarez AI, Verano L, García-Cabo C, Serrano-Pertierra E, Pascual J. Relationship between serum levels of VIP, but not of CGRP, and cranial autonomic parasympathetic symptoms: A study in chronic migraine patients. Cephalalgia. 2017; 37(9): 823-827.

10. Barbanti P, Fabbrini G, Pesare M, Vanacore N, Cerbo R. Unilateral cranial autonomic symptoms in migraine. Cephalalgia 2002; 22: 256–259.

11. Gulbenkian S, Uddman R, Edvinsson L. Neuronal messengers in the human cerebral circulation. Peptides. 2001; 22(6): 995–1007.

12. Jansen I, Uddman R, Ekman R, Olesen J, Ottosson A, Edvinsson L. Distribution and effects of neuropeptide Y, vasoactive intestinal peptide, substance P, and calcitonin gene-related peptide in human middle meningeal arteries: Comparison with cerebral and temporal arteries. Peptides. 1992; 13(3): 527–36.

13. Harmar AJ, Fahrenkrug J, Gozes I, Laburthe M, May V, Pisegna JR, Vaudry D, Vaudry H, Waschek JA, Said SI. Pharmacology and functions of receptors for vasoactive intestinal peptide and pituitary adenylate cyclase-activating polypeptide: IUPHAR Review 1. Br J Pharmacol. 2012; 166 (1): 4–17.

14. Domschke S, Domschke W, Bloom SR, Mitznegg P, Mitchell SJ, Lux G, et al. Vasoactive intestinal peptide in man: Pharmacokinetics, metabolic and circulatory effects. Gut. 1978; 19 (11): 1049–53.

15. Hansen JM, Sitarz J, Birk S, Rahmann AM, Oturai PS, Fahrenkrug J, Olesen J, Ashina M. Vasoactive intestinal polypeptide evokes only a minimal headache in healthy volunteers. Cephalalgia 2006; 26: 992-1003.

16. Gozes I, Fridkinb M, Hill JM, Brenneman DE. Pharmaceutical VIP: prospects and problems. Curr Med Chem. 1999; 6(11):1019–34.

17. Said SI. Vasoactive intestinal peptide. J Endocrinol Invest. 1986; 9 (2):191–200.

18. Frase LL, Gaffney FA, Lane LD, Buckey JC, Said SI, Blomqvist CG, et al. Cardiovascular effects of vasoactive intestinal peptide in healthy subjects. Am J Cardiol. 1987; 60 (16):1356–61.

19. Ottesen B, Gerstenberg T, Ulrichsen H, Manthorpe T, Fahrenkrug J, Wagner G. Vasoactive intestinal polypeptide (VIP) increases vaginal blood flow and inhibits uterine smooth muscle activity in women. Eur J Clin Invest. 1983; 13(4):321–4.

20. Morice AH, Sever PS. Vasoactive intestinal peptide as a bronchodilator in severe asthma. Peptides. 1986; 7 Suppl 1:279–80.

21. Calam J, Yiangou Y, Nikou GC, Chrysanthou BJ, Beacham JL, Bloom SR. Effects of preprovasoactive intestinal polypeptide-derived peptides on ileal output. Gastroenterology. 1990; 98(2): 505–8.

22. Kane MG, O'Dorisio TM, Krejs GJ. Production of secretory diarrhea by intravenous infusion of vasoactive intestinal polypeptide. N Engl J Med. 1983; 309 (24): 1482-1485.

23. Krejs GJ, Fordtran JS, Fahrenkrug J, Schaffalitzky de Muckadell OB, Fischer JE, Humphrey CS, et al. Effect of VIP infusion in water and ion transport in the human jejunum. Gastroenterology. 1980; 78(4):722–727.

24. Nyberg B, Einarsson K, Sonnenfeld T. Evidence that vasoactive intestinal peptide induces ductular secretion of bile in humans. Gastroenterology. 1989; 96 3):920–924.

25. Ottesen B, Pedersen B, Nielsen J, Dalgaard D, Wagner G, Fahrenkrug J. Vasoactive intestinal polypeptide (VIP) provokes vaginal lubrication in normal women. Peptides. 1987; 8(5): 797–800.

26. Eriksson LS, Hagenfeldt L, Mutt V, Wahren J. Influence of vasoactive intestinal polypeptide (VIP) on splanchnic and central hemodynamics in healthy subjects. Peptides. 1989; 10(2):481–4.

27. Headache Classification Committee of the International Headache Society (IHS). The International Classification of Headache Disorders, 3rd edition. Cephalalgia 2018; 38(1):1–211.

28. Dhand NK, Khatkar MS. Statulator: An online statistical calculator. Sample Size Calculator for Comparing Two Paired Means. 2014. Accessed at http://statulator.com/SampleSize/ss2PM.html.

29. Juto JE, Hallin RG. Kinetic oscillation stimulation as treatment of acute migraine: a randomized, controlled pilot study. Headache 2015; 55: 117-127.

30. Juto JE, Axelsson M. Kinetic oscillation stimulation as treatment of non-allergic rhinitis: an RCT study. Acta Oto Laryngologica 2014; 134: 506-512.

31. Möller M, Haji AA, Hoffmann J, May A. Peripheral provocation of cranial autonomic symptoms is not sufficient to trigger cluster headache attacks. Cephalalgia 2018; 38(8): 1498-1502.

32. Möller M, Schroeder CF, May A. Vagus nerve stimulation modulates the cranial trigeminal autonomic reflex. Ann Neurol. 2018; 84 (6): 886-892.

Appendix D: Hovedpineskema (infusionsfase), version 1.0, 01-10-2019

VIP/placebo, migrænepatienter uden aura

PT. NR:	INIT:	DATO:	START kl.:

TID		-10	0	10	20	30	40	50	60	70	80	90	100	110	120
Hovedpine	Styrke 0- 10														
Karakter	Dunkende														
	Trykkende														
	Jagende<br>Andet:														
Forv. v. hos.	Ja														
	Nej														
Som tidl opl.	Ja														
	Nej														
Kvalme	Ingen														
	Let														
	Middel														
	Svær														
Opkast	Ja														
	Nej														
Lysfølsom	Ingen														
	Let														
	Middel														
	Svær														
Blodtryk<br>MAP	Sys / Dia														
Puls															

Appendix D

PT. NR: INIT: DATO: START kl.:

TID		-10	0	10	20	30	40	50	60	70	80	90	100	110	120
Lydfølsom	Ingen														
	Let														
	Middel														
	Svær														
Lok	Ve. front														
	Ve. parietal														
	Ve. occipital														
	Ve. temporal														
	Ve. vertex														
	Ve. halvside														
	Hø front														
	Hø. parietal														
	Hø. occipital														
	Hø. temporal														
	Hø. vertex														
	Hø. halvside														
	Diffus														
Flushing	Ja														
	Nej														
Hjertebanken	Ja														
	Nej														
Varmeforn.	Ja														
	Nej														
Andet	Ja														
	Nej														
Dermascan															

Appendix D

VIP/placebo, Glostrup 2018/2019

PT. NR:	INIT:	DATO:	START kl.:

TID		130	140	150	160	170	180	190	200
Hovedpine	Styrke 0- 10								
Karakter	Dunkende								
	Trykkende								
	Jagende<br>Andet:								
Forv. v. hos.	Ja								
	Nej								
Som tidl opl.	Ja								
	Nej								
Kvalme	Ingen								
	Let								
	Middel								
	Svær								
Opkast	Ja								
	Nej								
Lysfølsom	Ingen								
	Let								
	Middel								
	Svær								
Blodtryk<br><br>MAP	Sys / Dia								
Puls									

Appendix D

PT. NR: INIT: DATO: START kl.:

TID		130	140	150	160	170	180	190	200
Lydfølsom	Ingen								
	Let								
	Middel								
	Svær								
Lok	Ve. front								
	Ve. parietal								
	Ve. occipital								
	Ve. temporal								
	Ve. vertex								
	Ve. halvside								
	Hø front								
	Hø. parietal								
	Hø. occipital								
	Hø. temporal								
	Hø. vertex								
	Hø. halvside								
	Diffus								
Flushing	Ja								
	Nej								
Hjertebanken	Ja								
	Nej								
Varmeforn.	Ja								
	Nej								
Andet	Ja								
	Nej								
Dermascan									

Appendix E: Hovedpineskema (efterfasen), version 1.0, 01/10/2019

Hjemmerapporteringsskema, hovedpine (5-12 timer efter indgift af VIP/placebo)

Skemaet udfyldes hver time efter undersøgelsen indtil sengetid. Der skal sættes kryds i felterne.

**Deltager-ID:**	**START kl.:**	**DATO:**

**Tid (timer efter indgift):**<br>**Klokkeslæt:**		**5**	**6**	**7**	**8**	**9**	**10**	**11**	**12**	
**Hovedpinens styrke?**<br>(0=ingen smerte, 10=værste smerte)	0 - 10									
**Lokalisation af hovedpine:**	■ Ve. pande									
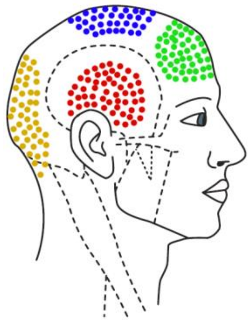	■ Ve. tinding									
	■ Ve. nakke									
	Ve. halvside									
	■ Hø. pande									
	■ Hø. tinding									
	■ Hø. nakke									
	Hø. halvside									
	■ Hovedtop									
	Diffus									
**Er hovedpinen**	Trykkende									
	Dunkende									
**Forværres hovedpinen ved fysisk aktivitet?**	Ja									
	Nej									
**Er der kvalme?**<br>(Sæt O hvis der er opkast)	Ja									
	Nej									
**Er du lysfølsom**	Ja									
	Nej									
**Er du lydfølsom**	Ja									
	Nej									
**Blev der taget medicin**<br>(angiv venligst navn og dosis nederst)	Ja									
	Nej									
**Har du syns- eller føle-forstyrrelser (aura)?**	Ja									
	Nej									
**Har du bemærket:**	Ualmindelig træthed									
	Stivhed i nakken									
	Gabetrang									
	Humørudsving									
	Koncentrationsbesvær									
	Sult									
	Tørst									
	Rødme									
	Varmefornnemmelser									
	Hjertebanken									
**Andet**<br>(beskriv andet på bagsiden)	Ja									
	Nej									

Medicin navn og dosis:	

Deltager-ID: **START kl.:** **DATO:**

Tid	Andet
5	
6	
7	
8	
9	
10	
11	
12	

Appendix G: Cranial Autonomic Parasympathetic Symptoms (CAPS) questionnaire
version 1.0, 01/10/2019

Deltager-ID:	Start:		Date:
**Symptom**	**Baseline**	**Infusion (0-120 min)**	**Post-infusion (120 min-12 hr)**
Lacrimation			
Conjunctival injection			
Eyelid edema			
Sensation of fullness in the ear			
Nasal congestion and/or rhinorrhea			

Legend: 0: absent; 1: present but mild; 2: present and conspicuous.
