# Supplementary material for: Effect of Vasoactive Intestinal Polypeptide on Development of Migraine Headaches: A Randomized Clinical Trial
Source: JAMA Netw Open. 2021 Aug 6;4(8):e2118543. doi: 10.1001/jamanetworkopen.2021.18543 (PMC8346940; doi:10.1001/jamanetworkopen.2021.18543)
Supplement: Supplement 2. — eTable 1. Headache-Associated Symptoms Recorded and Reported During Infusion and Postinfusion Periods eTable 2. Most Common Adverse Events Recorded During the Hospitalization Period eFigure 1. Localization of Headache During the Migraine-Like Attacks Induced by 2-Hour Infusion of VIP Compared With Spontaneous Migraine eFigure 2. Haemodynamic Effects of VIP [file jamanetwopen-e2118543-s002.pdf]

Supplemental Online Content

Pellesi L, Al-Karagholi MAM, De Icco R, et al. Effect of vasoactive intestinal polypeptide on development of migraine headaches: a randomized clinical trial. *JAMA Netw Open*. 2021;4(8):e2118543. doi:10.1001/jamanetworkopen.2021.18543

eTable 1. Headache-Associated Symptoms Recorded and Reported During Infusion and Postinfusion Periods

eTable 2. Most Common Adverse Events Recorded During the Hospitalization Period

eFigure 1. Localization of Headache During the Migraine-Like Attacks Induced by 2-Hour Infusion of VIP Compared With Spontaneous Migraine

eFigure 2. Haemodynamic Effects of VIP

This supplemental material has been provided by the authors to give readers additional information about their work.

© 2021 Pellesi L et al. *JAMA Network Open.*

eTable 1. Headache-Associated Symptoms Recorded and Reported During Infusion and Postinfusion Periods

	Incidence (n, %)	Time to onset	Duration
**Nausea**	18 (86%)	1 h 40 min (1 h 30 min – 1 h 45 min)	2 h 30 min (1 h 25 min – 4 h 30 min)
**Photophobia**	12 (57%)	45 min (20 min – 1 h 40 min)	3 h 30 min (50 min – 8 h 40 min)
**Phonophobia**	9 (43%)	1 h 10 min (40 min – 1 h 50 min)	3 h 5 min (50 min – 5 h 20 min)

n, %: number of subjects and percentages. Time to onset and duration are expressed as median and interquartiles.

© 2021 Pellesi L et al. *JAMA Network Open.*

eTable 2. Most Common Adverse Events Recorded During the Hospitalization Period[a]

	VIP (n = 21)	Placebo (n = 21)
Flushing	20	3
Warm sensations	20	2
Nausea	18	1
Heart palpitations	17	1
Photophobia	12	0
Cold sensations	12	0
Phonophobia	9	0
Abdominal discomfort	7	0
Back pain	5	0

[a]The hospitalization period consisted of 0 to 200 minutes.

© 2021 Pellesi L et al. *JAMA Network Open.*

eFigure 1. Localization of Headache During the Migraine-Like Attacks Induced by 2-Hour Infusion of VIP Compared

With Spontaneous Migraine

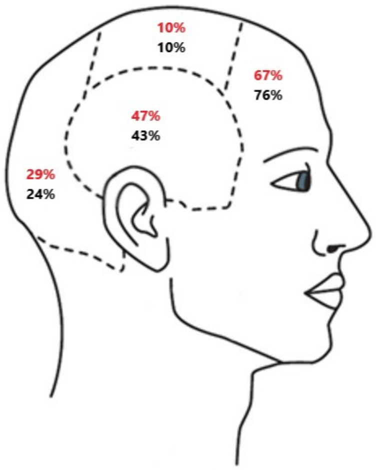

The percentages in red represent the percentages of patients who experienced headache in each location during migraine-like attacks induced by a 2-hour infusion of VIP, and the black percentages represent the localization of headache experienced with spontaneous migraine. The regions of the head are frontal, vertex (the upper surface of the head), temporal and occipital. VIP indicates vasoactive intestinal polypeptide.

© 2021 Pellesi L et al. *JAMA Network Open.*

eFigure 2. Haemodynamic Effects of VIP

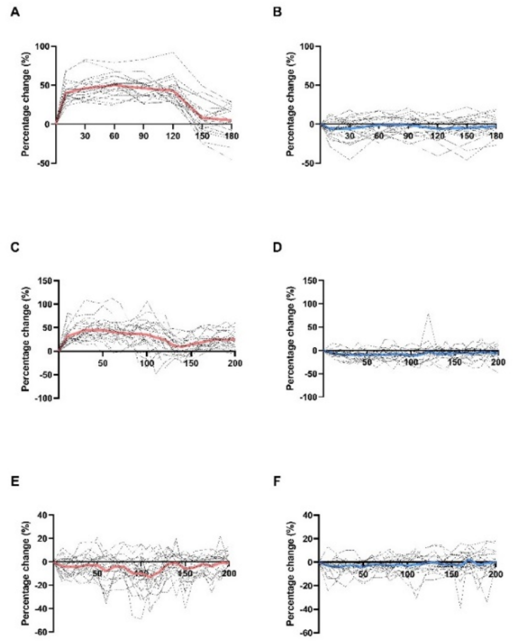

The abscissa refers to the time (minutes). (**A** and **B**) Mean (thick line) and individual (thin black lines) diameter of the superficial temporal artery (STA) after VIP (red) and placebo (blue). VIP significantly increased the STA diameter ($AUC_{0\text{-}180min}$, $p < 0.0001$) compared with placebo. (**C** and **D**) Mean (thick line) and individual (thin black lines) heart rate after VIP (red) and placebo (blue). VIP significantly increased the heart rate ($AUC_{0\text{-}200min}$, $p < 0.0001$) compared with placebo. (**E** and **F**) Mean (thick line) and individual (thin black lines) mean arterial pressure (MAP) after VIP (red) and placebo (blue). No significant difference was found regarding MAP ($AUC_{0\text{-}200min}$, $p = 0.728$) between VIP and placebo. VIP indicates vasoactive intestinal polypeptide.

© 2021 Pellesi L et al. *JAMA Network Open.*
