# Supplementary material for: Effect of Vasoactive Intestinal Polypeptide on Development of Migraine Headaches: A Randomized Clinical Trial
Source: JAMA Netw Open. 2021 Aug 6;4(8):e2118543. doi: 10.1001/jamanetworkopen.2021.18543 (PMC8346940; doi:10.1001/jamanetworkopen.2021.18543)
Supplement: Supplement 3. — Data Sharing Statement [file jamanetwopen-e2118543-s003.pdf]

Pellesi. Effect of Vasoactive Intestinal Polypeptide on Development of Migraine Headaches. *JAMA Netw Open*. Published August 06, 2021. doi:10.1001/jamanetworkopen.2021.18543

Data Sharing Statement

Data

Data available: Yes
Data types: Deidentified participant data
How to access data: Request for data must be sent to the corresponding author: ashina@dadlnet.dk.
When available: With publication

Supporting Documents

Document types: Informed consent form
How to access documents: Request for data must be sent to the corresponding author: ashina@dadlnet.dk.
When available: With publication

Additional Information

Who can access the data: Anyone upon reasonable request.
Types of analyses: For any purpose upon reasonable request.
Mechanisms of data availability: With a signed data access agreement.
